# Exploring the Potential of Eltrombopag: Room for More?

**DOI:** 10.3389/fphar.2022.906036

**Published:** 2022-05-23

**Authors:** Francesco Tarantini, Cosimo Cumbo, Luisa Anelli, Antonella Zagaria, Maria Rosa Conserva, Immacolata Redavid, Giorgina Specchia, Pellegrino Musto, Francesco Albano

**Affiliations:** ^1^ Department of Emergency and Organ Transplantation (D.E.T.O.), Hematology and Stem Cell Transplantation Unit, University of Bari “Aldo Moro”, Bari, Italy; ^2^ School of Medicine, University of Bari “Aldo Moro”, Bari, Italy

**Keywords:** eltrombopag, immune thrombocytopenia, severe aplastic anemia, myelodysplastic syndrome, acute myeloid leukemia, poor graft function

## Abstract

Since its introduction in clinical practice, eltrombopag (ELT) has demonstrated efficacy in heterogeneous clinical contexts, encompassing both benign and malignant diseases, thus leading researchers to make a more in-depth study of its mechanism of action. As a result, a growing body of evidence demonstrates that ELT displays many effects ranging from native thrombopoietin agonism to immunomodulation, anti-inflammatory, and metabolic properties. These features collectively explain ELT effectiveness in a broad spectrum of indications; moreover, they suggest that ELT could be effective in different, challenging clinical scenarios. We reviewed the extended ELT mechanism of action in various diseases, with the aim of further exploring its full potential and hypothesize new, fascinating indications.

## Introduction

Eltrombopag (ELT) is a thrombopoietin receptor agonist (TPO-RA), currently indicated for the treatment of immune thrombocytopenia (ITP), hepatitis C-associated thrombocytopenia and severe aplastic anemia (SAA) (Gilreath et al., 2021). Evidence shows that a subset of ITP patients achieving a complete response can safely suspend ELT without experiencing a relapse (González-López et al., 2015). Similarly, a trilineage response has been maintained in SAA patients after ELT discontinuation (Desmond et al., 2014).

Moreover, ELT has proven efficacy in myelodysplastic syndrome (MDS) and the therapy of post-transplant poor graft function (PGF) (Tang et al., 2018; Vicente et al., 2020), while data in the context of acute myeloid leukemia (AML) are conflicting (Buckstein, 2015; Mittelman et al., 2018; Frey et al., 2019; Shi et al., 2019). ELT can decrease cellular iron and enhance its mobilization in combination with available chelators, thus reducing reactive oxygen species (ROS) levels and improving cellular fitness in various clinical contexts (Roth et al., 2012; Vlachodimitropoulou et al., 2017; Argenziano et al., 2021).

These observations indicate that ELT exerts a complex mechanism of action beyond the stimulation of megakaryocytes proliferation, encompassing stem-cell stimulation, immunomodulation, and anti-inflammatory properties. The broad spectrum of indications and the increasing heterogeneity of reported effects suggest that ELT is able to set off a chain of events whose potential has yet to be fully exploited. We reviewed the main ELT related off-target effects in various clinical contexts to explore its potential: is there room for more?

## Mechanism of Action and Pharmacological Properties

**FIGURE 1:**
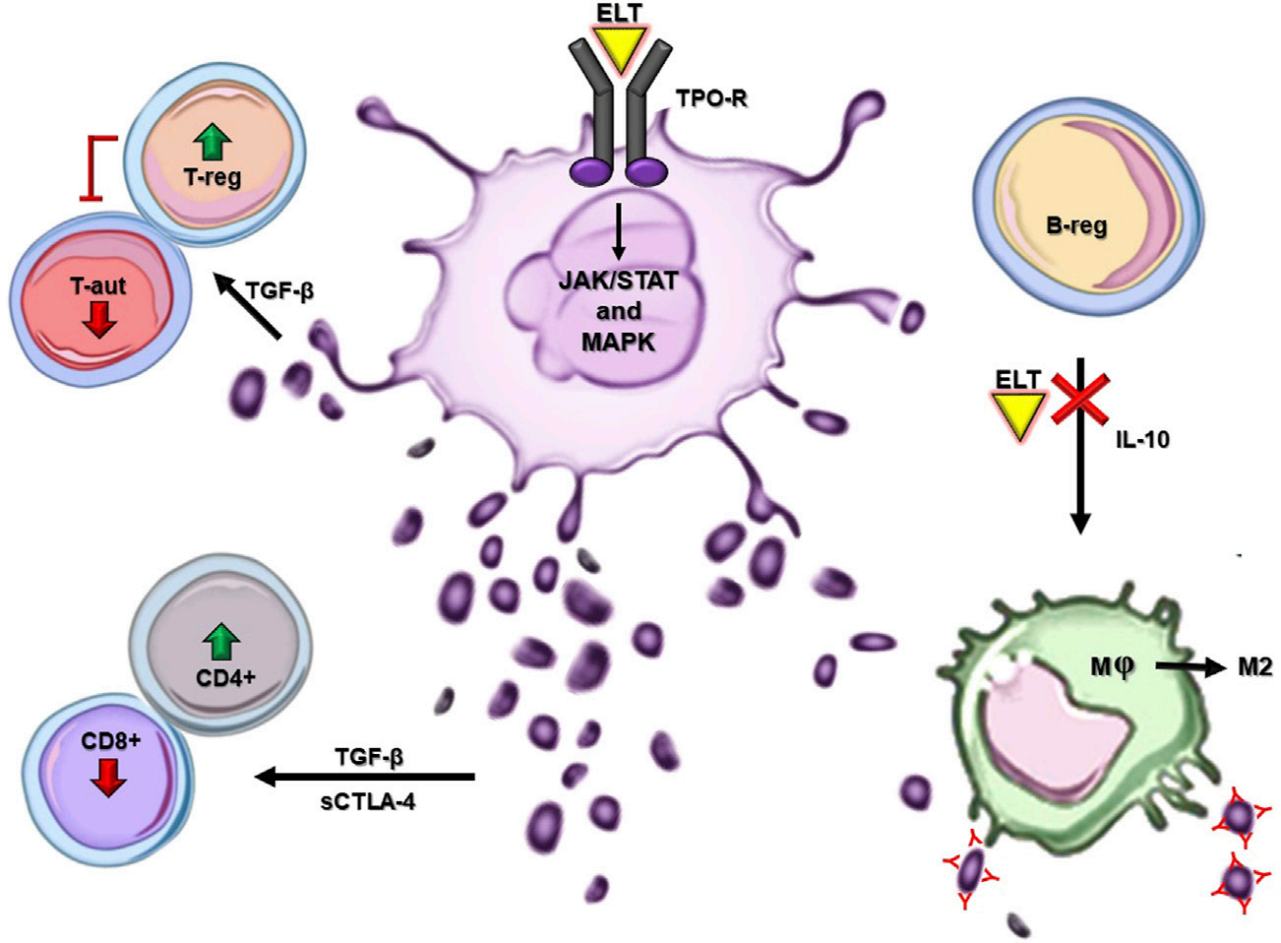
ELT mechanism of action and main off-target effects in ITP. Major cellular and molecular pathways exert the immunomodulatory and anti-inflammatory effects of ELT in ITP. In-silico models are not reported in this figure. ELT: eltrombopag, TPO-R: thrombopoietin receptor, T-reg: T regulatory lymphocyte, T-aut: T autoreactive lymphocyte, B-reg: B regulatory lymphocyte, CD4+: T helper lymphocyte, Cd8+: T cytotoxic lymphocyte, M: macrophage, M2: anti-inflammatory macrophage.

ELT is an oral synthetic nonpeptide low molecular weight TPO-RA. After administration, global bioavailability peaks at 2–6 h (Erickson-Miller et al., 2009; Gilreath et al., 2021). It circulates bound to plasma proteins, is metabolized in the liver through cytochrome P450 isoenzymes CYP1A, CYP2C8, and uridine diphosphate glucuronosyltransferase, and its half-life ranges from 21 to 32 h (Deng et al., 2011; Wire et al., 2012). Unlike endogenous TPO, ELT selectively binds to the transmembrane domain of the TPO receptor, stimulating survival, proliferation, and differentiation of megakaryocytes through the JAK/STAT and MAPK pathways (Figure 1) (Erickson-Miller et al., 2009). ELT activates STAT3/5, AKT, and ERK at a higher level than TPO, thus enabling the upregulation of megakaryopoiesis (di Buduo et al., 2016). As a result, ELT has an additive rather than a competitive effect on TPO, making it effective even in patients with high endogenous TPO levels. Evidence demonstrates that ELT displays a more extended spectrum of effects. Given its proven efficacy in treating heterogeneous clinical contexts, a growing body of evidence is unveiling ELT properties concerning the hematopoietic milieu, the immune system (IS), and the inflammation process.

## ITP: Drawing the Perfect Picture

ELT is indicated for ITP patients > 1-year-old refractory to first-line therapy or splenectomy (Gómez-Almaguer, 2018); its efficacy ranges between 75 and 95% (Gómez-Almaguer, 2018). Robust evidence supports the safety and success of dose tapering, re-treatment, and discontinuation of therapy; after this latter up to approximately 50% of patients maintain a response (Mahévas et al., 2014; González-López et al., 2015; Wong et al., 2017; Al-Samkari and Kuter, 2018; Ghanima et al., 2019). This unique profile has fueled interest in making a more in-depth study of the mechanism of action of ELT beyond TPO-R agonism. Interestingly, while binding only human and primate TPO-R, ELT has proven efficacy in murine models, thus reinforcing the idea of beneficial off-target effects (Erickson-Miller et al., 2009; Raslova et al., 2016).

Based on the physiopathological mechanisms of ITP, the prime suspect was the IS. Firstly, ELT is thought to exert an indirect immunomodulatory effect: by increasing the number of platelets, it should, on one hand, increase the number of CD4^+^ T helper cells and reduce the number of CD8^+^ T effector cells (Figure 1) (Bao et al., 2010; Liu et al., 2013). Since platelets are the main reservoir of TGF-β, this effect should partly be mediated by an increased activity of this anti-inflammatory cytokine (Abbonante et al., 2016); its expression has been demonstrated to be positively correlated with soluble CTLA-4 levels, which, in turn, can modulate the immune response (Fujita et al., 2012). TGF-β is also involved in the downregulation of autoreactive T cells and expansion of Tregs (Liu et al., 2018). Additionally, TGF-β is highly expressed in ectosomes: platelet-derived microparticles interacting with immune cells to restore IS homeostasis and dampen inflammation (Figure 1) (Sadallah et al., 2011, 2014). On the other hand, ELT should restore immune tolerance to platelet autoantigens, thus altering the ITP immunopathogenic process (Nishimoto et al., 2014). Moreover, upon ELT treatment in ITP, the TGF-β increased secretion and further fibrogenic cytokines are thought to be responsible for augmented reticulin bone marrow (BM) fibrosis. This process is reversible and tends to worsen in a time-dependent fashion. Furthermore, ELT administration seems to induce myeloproliferative neoplasm-like changes in the ITP BM, such as trilineage hypercellularity, and megakaryocytic pleomorphic proliferation together with the tendence to form clusters (Boiocchi et al., 2012; Ghanima et al., 2014; Brynes et al., 2017).

Moreover, evidence demonstrates that ELT treatment in ITP results in both quantitative and qualitative immunomodulatory effects, namely increased regulatory B-cell numbers and a reduced phagocytic capacity of monocyte-derived macrophages on opsonized platelets (Li et al., 2012; Liu et al., 2016). Furthermore, ELT may influence macrophages polarization towards the M2 anti-inflammatory subtype (Figure 1) (di Paola et al., 2021). Bearing in mind that a skewed proinflammatory T cell profile characterizes ITP, ELT has been shown to exert an antiproliferative effect on T cells and to affect their functionality, reducing the intracellular production of TNF-α, INF-γ and granzyme B (Sayed et al., 2019).

The consistency of all these data is even more accentuated considering that RNA sequencing of blood cells subsets has revealed that TPO is expressed in immune cell subpopulations: T-regs, B lymphocytes, CD4^+^ and CD8^+^ T cells, monocytes, neutrophils, and NK cells (Schmiedel et al., 2018; Monaco et al., 2019). Recently, an “in silico” approach has shown different pathways (upregulation of FOXP3 and PPARγ, attenuation of IFN-γ signaling) by which ELT may restore immune tolerance and reduce inflammation in the context of ITP (Lozano et al., 2021). Interestingly, this model hypothesizes a potential pro-apoptotic BCL-2 mediated effect upon long term treatment with ELT, affecting the survival of platelets (Lozano et al., 2021). On the other hand, experimental data show that ELT could directly inhibit the pro-apoptotic factor Bax, thus contributing to the fine regulation of cell survival pathways (Spitz et al., 2021). It remains to be elucidated whether the same effects may occur at the lymphocyte level, ultimately affecting immunomodulation. In this scenario, the body of evidence arising from ITP suggests that the effectiveness of ELT in various clinical contexts on multiple routes should be explored.

## SAA: Unlock the Stemness

The rationale for using ELT in SAA lies in the beneficial effect of TPO on the expansion of hematopoietic stem cells (HSCs) (Scheinberg, 2018; Drexler and Passweg, 2021). There is evidence that HSCs and immature progenitors express TPO-R on their cell surface (Ninos et al., 2006; de Graaf and Metcalf, 2011); ELT-mediated stimulation of TPO-R downstream pathways can, therefore, effectively “unlock” bi- or tri-lineage hemopoiesis (Olnes et al., 2012). Interestingly, a sustained hematologic response has been observed in SAA patients after ELT discontinuation, thus raising questions about possible beneficial off-target effects leading to efficient long-term maintenance of HSCs fitness and proliferation (Desmond et al., 2014).

The IS dysregulation is a cornerstone of the SAA pathogenesis, characterized by a clonal expansion of CD8^+^ T cytotoxic cells and a reduced number of dysfunctional T-regs (Schoettler and Nathan, 2018; Young, 2018). Moreover, T-helper 1, 2 and 17 subsets are also increased, further supporting the effector response and a proinflammatory state sustained by IL-2, TNF-α and IFN-γ (Peffault De Latour et al., 2010; Medinger et al., 2018). It is plausible that the effects mentioned above observed in ITP could also contribute to ELT effectiveness in SAA.

Furthermore, it has been shown that ELT can overcome the inhibitory action exerted by IFN-γ on the TPO-TPO-R axis (Alvarado et al., 2019). IFN-γ, in fact, prevents the full binding of TPO explicitly to TPO-R through steric occlusion of the binding site, thus affecting downstream signaling pathways, resulting in a decreased survival of HSCs. Thanks to its allosteric binding site, ELT bypasses this inhibition, explaining its clinical activity even in the presence of high endogenous TPO levels, such as in SAA and BM failure (Alvarado et al., 2019).

Notably, robust evidence supports ELT iron chelation properties that could ameliorate the fitness of HSCs and hematopoiesis, especially considering that SAA patients are frequently transfusion-dependent (Vlachodimitropoulou et al., 2017; Zhao et al., 2018). Moreover, the ELT-mediated mobilization of intracellular iron and the decrease of serum ferritin levels observed in various clinical contexts (Roth et al., 2012; Vlachodimitropoulou et al., 2017; Punzo et al., 2018; Zhao et al., 2018; Argenziano et al., 2021) could help to prevent long-term multi-organ complications and improve BM function.

Based on this knowledge, the efficacy of ELT in the treatment of SAA can be considered multifactorial (Table 1). The safety of treatment discontinuation seems to suggest the presence of other mechanisms accounting for the ELT success, which have yet to be fully elucidated.

**TABLE 1:** ELT effects in diseases other than ITP.

Disease	ELT effects	References
SAA	• bi- or tri- lineage hemopoiesis induction	(Olnes et al. (2012)) (Alvarado et al. (2019)) (Vlachodimitropoulou et al. (2017); Zhao et al. (2018)) (Roth et al. (2012); Vlachodimitropoulou et al. (2017); Punzo et al. (2018); Zhao et al. (2018); Argenziano et al. (2021)) (Peffault De Latour et al. (2010); Medinger et al. (2018); Schoettler and Nathan, (2018); Young, (2018))
	• HSCs survival promotion (removing INF-γ inhibition on the TPO-TPO-R axis)	
	• HSCs fitness improvement (through iron chelation)	
	• recovery of BM fitness (through serum ferritin levels reduction)	
	• T helper cells increase and T effector cells reduction as in ITP?	
MDS and AML	• megakaryopoiesis improvement	(Platzbecker et al. (2015)) (Roth et al. (2012); Argenziano et al. (2021)) (Kalota et al. (2015)) (Roth et al. (2012); Argenziano et al. (2021)) (Olnes et al. (2012); Babushok, (2018))
	• antileukemic effects related to iron chelation	
	• pro-apoptotic signaling activation through ROS modulation	
	• cellular differentiation induction	
	• clonal evolution induction as in SAA?	
PGF	• impaired hematopoiesis rescue	(Marotta et al. (2019); Yuan et al. (2019)) (Vogel et al. (2019)) (Penack et al. (2020))
	• human CMV replication inhibition	
	• recovery of BM fitness (through serum ferritin levels reduction)	
	• possible use in GvHD without affecting GvL?	

ITP, Immune thrombocytopenia; ELT, Eltrombopag; SAA, Sever aplastic anemia; MDS, Myelodysplastic syndrome; AML, Acute myeloid leukemia; PGF, Poor graft function; HSCs, Hematopoietic stem cells; BM, bone marrow; ROS, Reactive oxygen species; CMV, Cytomegalovirus; GvHD, Graft versus host disease; GvL, Graft versus leukemia.

## MDS and AML: A Dangerous Path

In the wake of the encouraging results in ITP and SAA, various studies tested ELT efficacy in MDS and AML (Buckstein, 2015; Platzbecker et al., 2015; Dickinson et al., 2018; Tang et al., 2018; Shi et al., 2019; Vicente et al., 2020). Beyond the need for improvement of megakaryopoiesis in both diseases, these attempts were based on founding concepts such as the IS dysregulation shared by SAA and the subset of hypoplastic MDS (Calado, 2011), and the ELT capacity to influence HSCs fitness and the BM microenvironment, and to restore “compromised” hematopoiesis. On the other hand, the main concern was the risk of clonal evolution upon ELT treatment, as previously observed in SAA patients (Table 1) (Olnes et al., 2012; Babushok, 2018). In detail, approximately 15–20% of SAA patients have been demonstrated to develop clonal cytogenetic BM abnormalities (including chromosome 7 aberrations) early upon initiation of treatment with ELT (Winkler et al., 2019). On the other hand, various studies demonstrated that in SAA the presence of mutations in myeloid candidate genes is not sufficient for malignant transformation and weakly predictive of clonal evolution (Winkler et al., 2019). Therefore, it should be noted that there is no consensus as to the exact mechanism of ELT-mediated clonal evolution in SAA. Moreover, preliminary studies aiming to evaluate ELT efficacy and safety in the context of MDS and AML did not show increased blast proliferation or clonogenic properties, while confirming the improvement in platelets counts (Will et al., 2009; Mavroudi et al., 2011). Furthermore, ELT showed antileukemic effects related to its iron chelation properties, the capacity to activate pro-apoptotic signaling through reactive oxygen species (ROS) modulation and induce cellular differentiation (Roth et al., 2012; Kalota et al., 2015; Argenziano et al., 2021). Against this background, results from MDS and AML trials confirm ELT effectiveness in treating hypoplastic and low to intermediate-1 risk MDS, while data from higher-risk MDS and AML are conflicting and affected by the rates of clonal evolution and disease progression (Buckstein, 2015; Mittelman et al., 2018; Frey et al., 2019; Vicente et al., 2020). These findings are of great interest, prompting a reflection on the possible role of ELT off-target effects in hematological malignancies. Considering that preclinical evidence supports no direct ELT detrimental effects on blasts proliferation and clonal evolution in both MDS and AML, data from clinical trials challenge this concept (Platzbecker et al., 2015; Dickinson et al., 2018). We may hypothesize that the above-mentioned ELT effects on the IS could affect the antitumoral immune surveillance and the cytotoxic response, making the leukemic cell’s life much easier; moreover, the concept that dampening the IS could favor the emergence of new clones, contributing to the disease activity, should be further investigated. On the other hand, considering that a proinflammatory state favors the proliferation of mutated HSCs despite normal ones (Lusis, 2021), the anti-inflammatory environmental changes induced by ELT could somehow compensate for the IS attenuation and lack of immune surveillance. This intricate scenario should take into account the interplay between ELT and apoptotic signaling (demonstrated, on one hand, by its interaction with Bax and, on the other hand, by the ROS modulation affecting leukemic cells metabolism) and the ELT-mediated reduction of intracellular iron levels inducing cellular differentiation and the inhibition of blast proliferation.

## PGF: Keeping the Balance

PGF occurs in up to 20% of patients; it is defined by the presence of persistent cytopenias (mainly thrombocytopenia) after completion of allo-HSCT procedures. The physiopathology of PGF is not fully understood; factors such as the graft composition, HLA mismatches, conditioning regimen and immunosuppression, graft versus host disease (GvHD), post-transplant infections and viral reactivations are implicated in the PGF pathogenesis (Chen et al., 2020).

Given the lack of effective treatments and considering ELT-induced sustained multilineage response in SAA, various studies evaluated ELT efficacy in PGF, showing encouraging results (Table 1) (Dyba et al., 2016; Marotta et al., 2019; Yuan et al., 2019; Halahleh et al., 2021). Again, beyond TPO agonism, direct HSCs stimulation, immunomodulation, and anti-inflammatory properties are considered the main mechanisms behind the ELT capacity to rescue impaired hematopoiesis in allo-HSCT patients and revert PGF (Marotta et al., 2019; Yuan et al., 2019). Notably, ELT efficacy seems to be irrespective of the putative PGF cause (Marotta et al., 2019). It is noteworthy that ELT is capable of inhibiting human cytomegalovirus (CMV) replication through its iron chelation properties (Vogel et al., 2019); this is of great importance, considering that CMV reactivation is one of the most typical causes of PGF (Chen et al., 2020).

Taken together, these data do not only confirm that ELT can be safely used to treat PGF by different routes (Table 1) but also prompt a reflection on its possible use in the intricate context of GvHD, where immune dysregulation and a generalized proinflammatory state are of pivotal importance (Cooke et al., 2017; Hess et al., 2021). On one hand, we should consider that ELT anti-inflammatory properties, the modulation of cytotoxic response and restoration of iron overload could be helpful in the attenuation of GvHD; on the other, robust evidence underlines the importance of TGF-β signaling in the dynamics of GvHD (Carli et al., 2012). In particular, this master regulator of the IS has been shown to inhibit the development of acute-GvHD and, conversely, to contribute to end-stage organ fibrosis in chronic-GVHD (Carli et al., 2012). Furthermore, the inhibition of TGF-β signaling is thought to positively influence the graft versus leukemia effect (GvL) (Carli et al., 2012). Hence, we should consider the impact of ELT on TGF-β homeostasis; we have previously discussed the interplay between ELT and TGF-β as described in the context of ITP, bearing in mind the physiological role of platelets as the TGF-β reservoir.

Moreover, considering that allo-HSCT patients frequently exhibit high serum ferritin levels and that iron overload affects their prognosis, ELT iron chelation properties could be helpful in the recovery of BM microenvironment fitness, ameliorating their clinical course (Penack et al., 2020). Considering the ELT-related risk of clonal evolution and leukemic cells proliferation, studies are needed to investigate whether the ELT range of beneficial effects in PGF could extend to treating acute GvHD without affecting GvL.

## Discussion

Since its introduction in clinical practice, ELT has proven to be efficacious in heterogeneous contexts. Unexpected results in both immune-mediated, benign, and malignant diseases prompted more in-depth study of its mechanisms of action, such that nowadays, TPO agonism, immunomodulation anti-inflammatory and metabolic properties are considered the cornerstones of ELT efficacy. This broad spectrum of effects has led researchers to test ELT in various clinical contexts, with encouraging results. Nevertheless, the increasing knowledge of the IS importance and microenvironmental factors in both the onset and the course of hematological diseases suggests that ELT use should be explore in ever more scenarios to fully exploit its potential: there could be room for more.

## Data Availability

The original contributions presented in the study are included in the article/Supplementary material, further inquiries can be directed to the corresponding author.
